# Supplementary figures and images for: Anti-leukemic effect of menthol, a peppermint compound, on induction of apoptosis and autophagy
Source: PeerJ. 2023 Mar 10;11:e15049. doi: 10.7717/peerj.15049 (PMC10010179; doi:10.7717/peerj.15049)

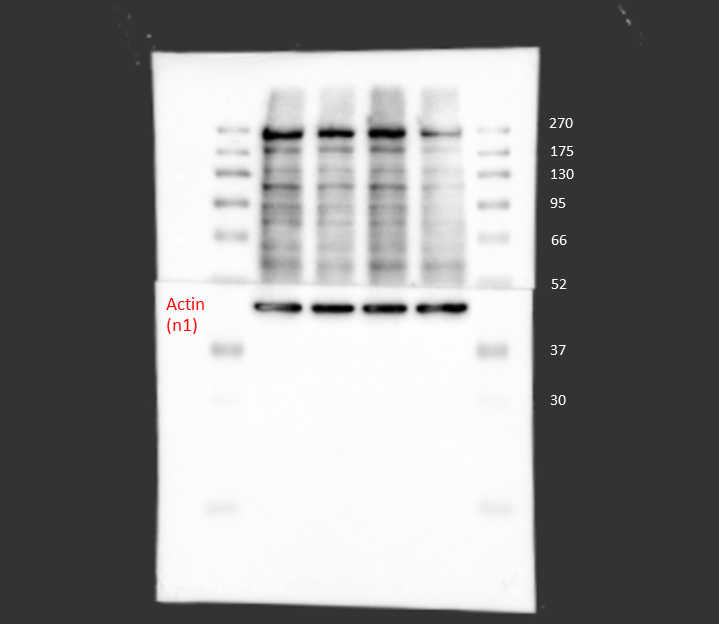

Supplement: Supplemental Information 2 [file peerj-11-15049-s002.zip › Uncropped blots/Casp3/(n1) Actin.png]

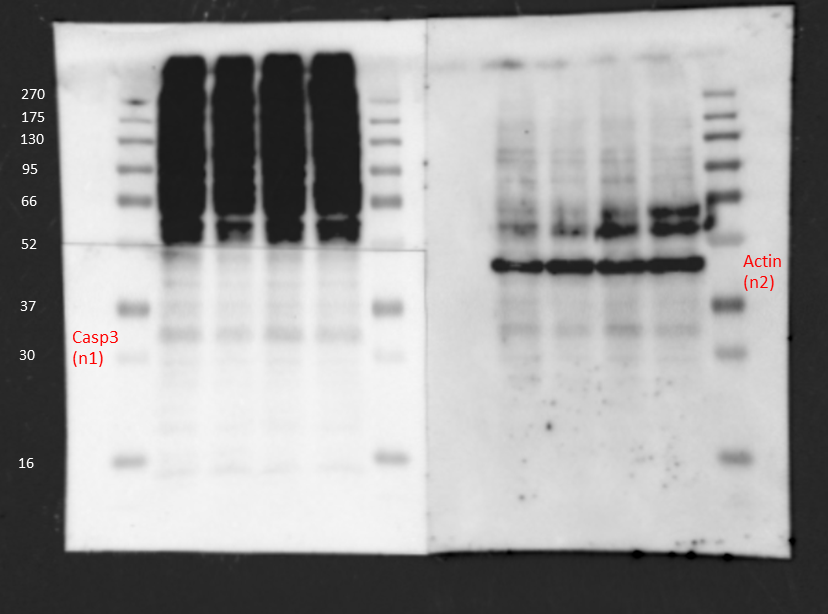

Supplement: Supplemental Information 2 [file peerj-11-15049-s002.zip › Uncropped blots/Casp3/(n1) Casp3 and (n2) Actin.png]

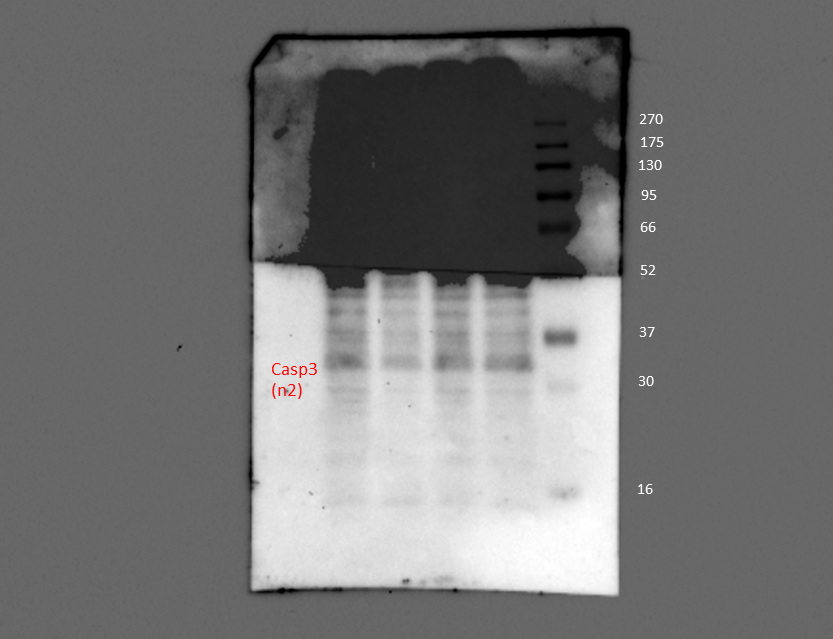

Supplement: Supplemental Information 2 [file peerj-11-15049-s002.zip › Uncropped blots/Casp3/(n2) Casp3.png]

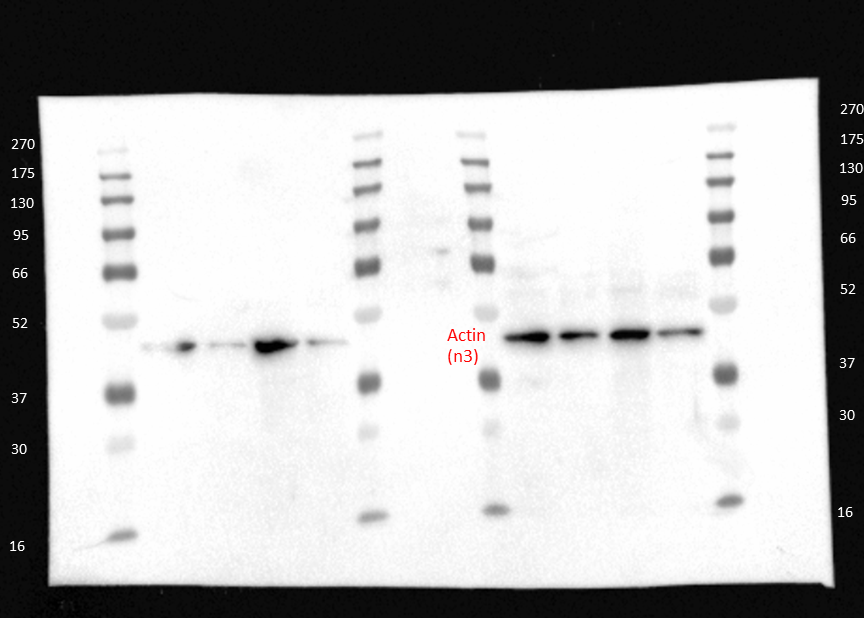

Supplement: Supplemental Information 2 [file peerj-11-15049-s002.zip › Uncropped blots/Casp3/(n3) Actin.png]

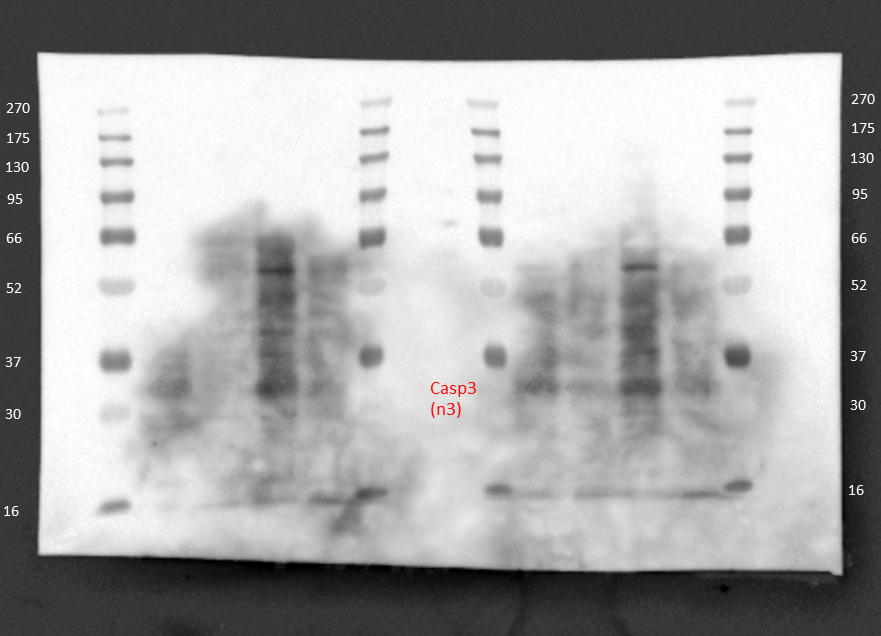

Supplement: Supplemental Information 2 [file peerj-11-15049-s002.zip › Uncropped blots/Casp3/(n3) Casp3.png]

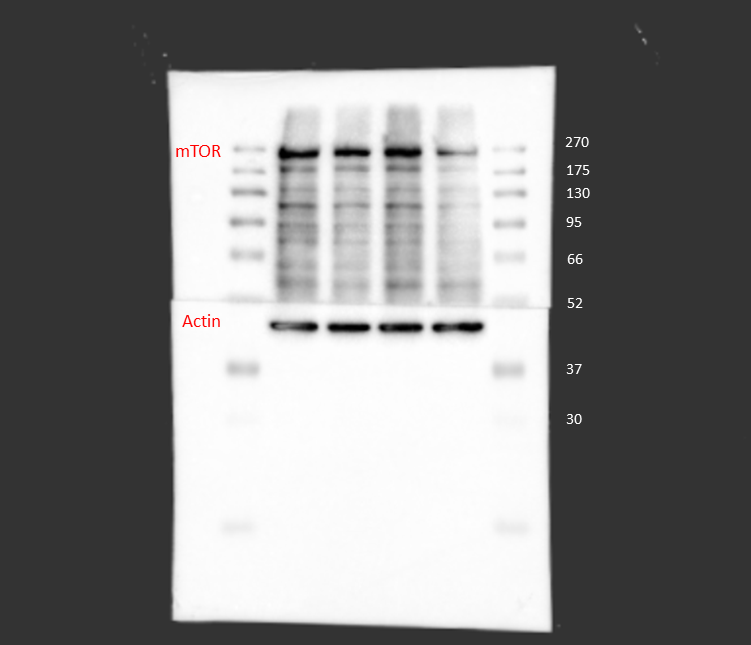

Supplement: Supplemental Information 2 [file peerj-11-15049-s002.zip › Uncropped blots/mTOR/(n1) mTOR and Actin.png]

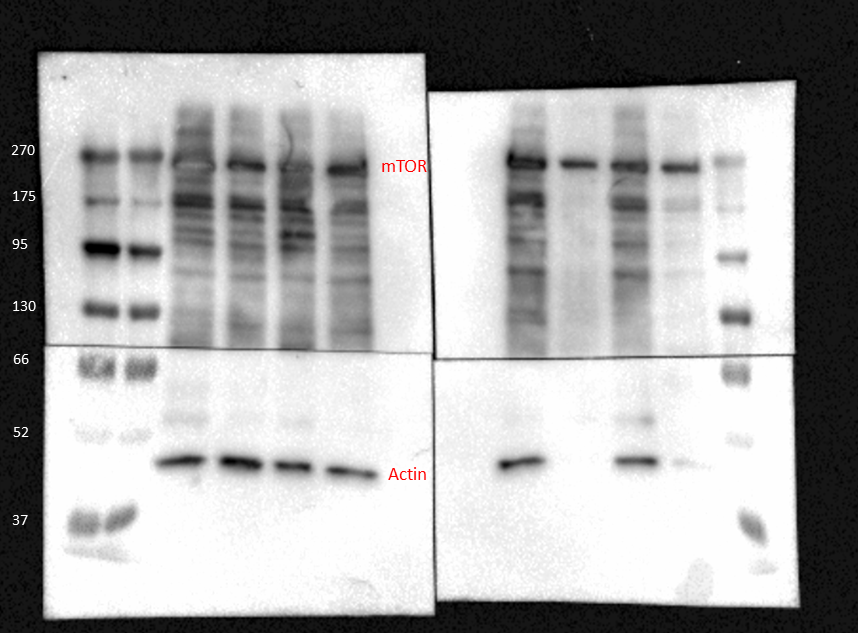

Supplement: Supplemental Information 2 [file peerj-11-15049-s002.zip › Uncropped blots/mTOR/(n2) mTOR and Actin.png]

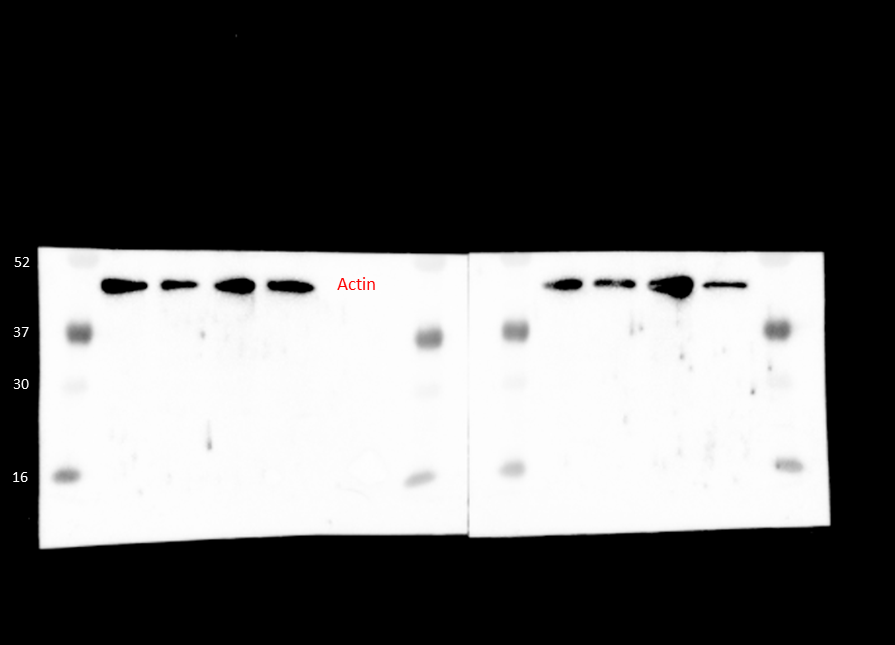

Supplement: Supplemental Information 2 [file peerj-11-15049-s002.zip › Uncropped blots/mTOR/(n3) Actin.png]

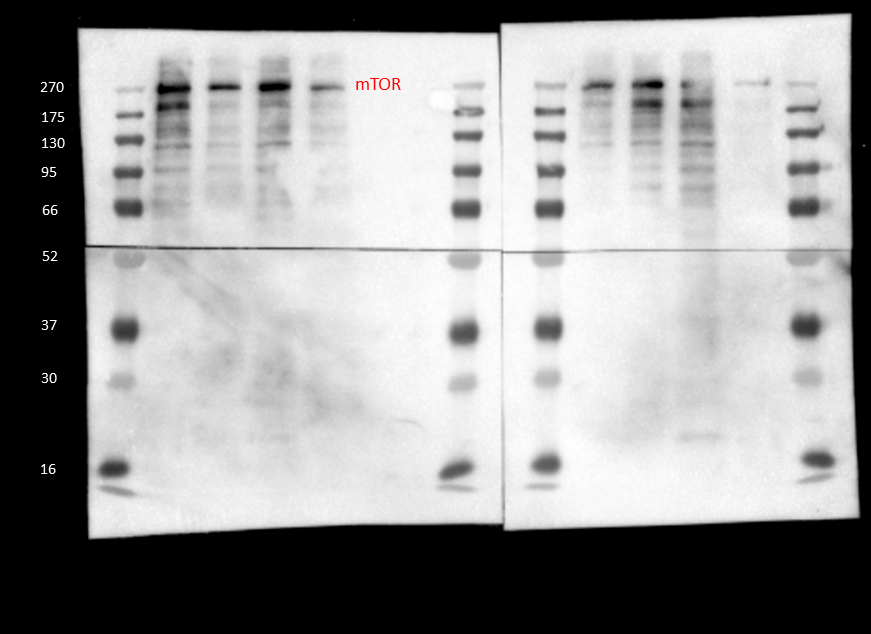

Supplement: Supplemental Information 2 [file peerj-11-15049-s002.zip › Uncropped blots/mTOR/(n3) mTOR.png]
